# Bile acid toxicity in Paneth cells contributes to gut dysbiosis induced by high-fat feeding

**DOI:** 10.1172/jci.insight.138881

**Published:** 2020-10-15

**Authors:** Hui Zhou, Shi-Yi Zhou, Merritt Gillilland, Ji-Yao Li, Allen Lee, Jun Gao, Guanpo Zhang, Xianjun Xu, Chung Owyang

**Affiliations:** 1 Division of Gastroenterology and Hepatology, Department of Internal Medicine, University of Michigan Health System, Ann Arbor, Michigan, USA.; 2 Department of Gastroenterology, Shanghai General Hospital, Shanghai Jiao Tong University School of Medicine, Shanghai, China.; 3 Department of Gastroenterology, 900 Hospital of the Joint Logistics Team, Fuzhou, China.

**Keywords:** Gastroenterology, Defensins, Obesity

## Abstract

High-fat feeding (HFF) leads to gut dysbiosis through unclear mechanisms. We hypothesize that bile acids secreted in response to high-fat diets (HFDs) may act on intestinal Paneth cells, leading to gut dysbiosis. We found that HFF resulted in widespread taxonomic shifts in the bacteria of the ileal mucosa, characterized by depletion of *Lactobacillus* and enrichment of *Akkermansia muciniphila*, *Clostridium* XIVa, Ruminococcaceae, and Lachnospiraceae, which were prevented by the bile acid binder cholestyramine. Immunohistochemistry and in situ hybridization studies showed that G protein–coupled bile acid receptor (TGR5) expressed in Paneth cells was upregulated in the rats fed HFD or normal chow supplemented with cholic acid. This was accompanied by decreased lysozyme^+^ Paneth cells and α-defensin 5 and 6 and increased expression of *XBP-1*. Pretreatment with ER stress inhibitor 4PBA or with cholestyramine prevented these changes. Ileal explants incubated with deoxycholic acid or cholic acid caused a decrease in α-defensin 5 and 6 and an increase in *XBP-1*, which was prevented by TGR5 antibody or 4PBA. In conclusion, this is the first demonstration to our knowledge that TGR5 is expressed in Paneth cells. HFF resulted in increased bile acid secretion and upregulation of TGR5 expression in Paneth cells. Bile acid toxicity in Paneth cells contributes to gut dysbiosis induced by HFF.

## Abstract 2

High fat feeding upregulates G protein coupled bile acid receptor (TGR5) in small intestinal Paneth cells, which may contribute to gut dysbiosis.

## Introduction

Chronic high-fat feeding (HFF) may induce gut dysbiosis and alter host homeostasis, resulting in mucosal inflammation and metabolic endotoxemia (1–3). However, little is known about how HFF induces gut dysbiosis. While HFF may contribute directly to gut dysbiosis (4, 5), it is also possible that alterations in gut bacterial communities may be mediated by host factors in response to high-fat diet (HFD).

HFF leads to an increase in circulating bile acids (6) and affects gut bacterial communities by changing the bile acid profile (7, 8). Bile acids recently have been reported to play a role in gut dysbiosis (9–11). After ingestion of a meal, the primary bile acids (cholic acid [CA] and chenodeoxycholic acid) are secreted into the duodenum, where they facilitate fat absorption. Normally, more than 95% are efficiently absorbed from the terminal ileum and transported back to the liver via the enterohepatic circulation. Small quantities of primary bile acids reach the terminal ileum and colon and undergo deconjugation and dehydroxylation by certain gut bacteria to form secondary bile acids, deoxycholic acid (DCA) and lithocholic acid (LCA) (12). Bile acids, especially secondary forms such as DCA, have strong antimicrobial activity and cytotoxicity and may regulate the composition of gut bacterial communities and host physiology (13, 14). In addition to the direct antimicrobial actions, we explored other mechanisms by which bile acids may affect host factors and alter the gut bacterial composition.

Both primary and secondary bile acids exert their effects by activating nuclear (15) and plasma membrane receptors (16, 17). G protein–coupled receptor TGR5 is a bile acid plasma membrane receptor that is widely distributed in the gastrointestinal tract (18). TGR5 regulates bile acid synthesis, intestinal secretion, glucose homeostasis, energy expenditure, gastrointestinal motility, and inflammation, while overstimulation has been linked to human diseases, such as esophageal and gastric carcinoma (19–23). Here, we hypothesis that TGR5 receptors are present in Paneth cells. This may provide a mechanism by which bile acids regulate intestinal immune function.

Paneth cells are highly specialized epithelial cells located at the base of the crypts of Lieberkühn of the small intestine. Paneth cells secrete antimicrobial peptides into the crypt lumen, which protect the host from enteric pathogens, help to shape the composition of the colonizing bacteria, safeguard against bacterial translocation across the epithelium, and act as immune modulators and trophic factors (24–26). On the other hand, Paneth cell dysfunction has been associated with gut bacterial changes in obese individuals (27). Recently, Zheng et al. reported that a HFD caused rapid and significant increases in the intestinal bile acid pool, accompanied by an alteration in bacterial composition (7). This raises the possibility that, in addition to the direct effect of bile acid on intestinal bacteria, bile acids may modulate gut immune function and indirectly alter gut bacterial communities. We hypothesized that bile acids elevated by HFF regulate intestinal Paneth cell function, which may contribute to gut dysbiosis in the ileal mucosa.

## Results

### TGR5 expression in the Paneth cells, and HFF increases bile acids and enhances TGR5 expression in ileal crypts.

**Figure 1:**
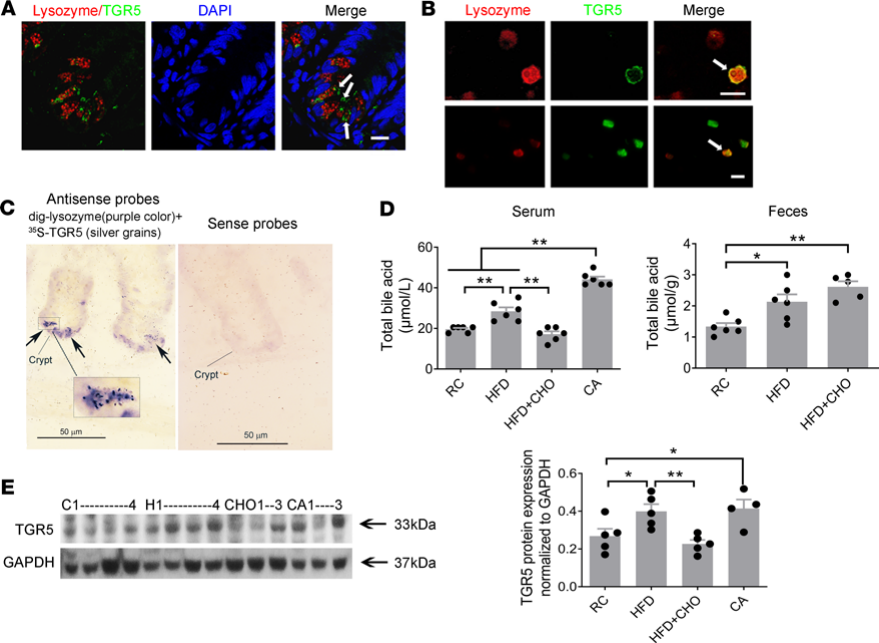
TGR5 expression in the Paneth cells; HFD increases bile acids and enhances TGR5 expression in ileal crypts. (**A** and **B**) Immunocytochemical staining using sections of ileal specimens and isolated crypt cells showed that TGR5 expression was observed in the lysozyme^+^ Paneth cells (white arrows) in ileal crypts. Scale bar: 10 μm. (**C**) Left: hybridization using antisense probes. Purple represents lysozyme mRNA, a specific marker for Paneth cells, which are located at the bottom portion of crypt; silver grains indicate TGR5 mRNA. Arrows indicate colocalization. Right: Dig-lysozyme and [^35^S]-TGR5 sense probes were used as the negative control. Note that TGR5 receptors are also expressed in other cell types in the intestinal crypts. Scale bar: 50 μm; 3-fold magnification (inset). (**D**) Measurement of total bile acid concentration. A 2-week high-fat diet (HFD) induced a 45% increase in serum and 60% increase in fecal bile acid compared with control (RC) (*n* = 5–6). The increased total serum bile acid was prevented by concurrent administration of cholestyramine (*n* = 6). A similar increase in serum bile acid was observed in cholic acid group (*n* = 6). (**E**) The density of the TGR5-immunoreactive band at 33 kDa was observed in the ileal crypts of rats given HFD. TGR5 protein expression increased in ileal crypts of HFD rats compared with control (*n* = 5). This increase was prevented by concurrent administration of cholestyramine (*n* = 5). A similar increase was also observed in rats with cholic acid (*n* = 4–5). Each bar represents mean ± SEM. C, RC; H, HFD; CHO, HFD + cholestyramine; CA, cholic acid. *P* values were determined by 1-way ANOVA. *P < 0.05, **P < 0.01.

Immunohistochemical staining was performed using ileal specimens and isolated crypt cells. TGR5 expression was observed in the ileal crypts. Lysozyme staining was used to identify Paneth cells. Merged images show that TGR5 was expressed by Paneth cells (Figure 1A). These findings were confirmed in isolated crypt cells. A lysozyme^+^ Paneth cell expressing TGR5 is shown in Figure 1B. Double in situ hybridization using dig-labeled lysozyme, a specific marker for Paneth cells, and [^35^S]-labeled TGR5 confirmed that 78% of Paneth cells expressed TGR5 (Figure 1C).

Rats fed a control diet of regular chow (RC, 11% kcal from fat) had a fecal bile acid level of 1.3 ± 0.1 μmol/g. A 2-week HFF (58% kcal from fat) increased the fecal bile acid level to 2.1 ± 0.2 μmol/g (*n* = 6, *P* < 0.05 compared with RC; Figure 1D). A similar increase was observed in rats fed a HFD supplemented with cholestyramine (CHO) (2.6 ± 0.2 μmol/g; *n* = 6, *P* < 0.01 compared with RC; Figure 1D). Rats fed RC had a serum bile acid level of 19.6 ± 0.6 μmol/L after a 12-hour fast. A 2-week HFF increased the fasting serum bile acid level to 28.4 ± 2.0 μmol/L (*n* = 6, *P* < 0.01; Figure 1D). A similar increase was observed in rats fed RC supplemented with CA (44.4 ± 1.4 μmol/L; *n* = 6, *P* < 0.01; Figure 1D). Serum bile acid levels were normalized by concurrent administration of CHO in rats on HFD (17.2 ± 1.4 μmol/L; *n* = 6, *P* < 0.01; Figure 1D). These results indicate that HFF elevates fecal and serum bile acid level.

Compared with that in controls, there was a significant increase in TGR5 protein expression in ileal crypts from rats fed a HFD (*n* = 5, *P* < 0.05) or RC supplemented with CA (*n* = 4, *P* < 0.05; Figure 1E). Cofeeding with CHO prevented the increase TGR5 in response to HFD (*n* = 5, *P* < 0.01; Figure 1E).

### HFF induces a decrease in Paneth cells and antimicrobial peptides, which is prevented by cofeeding with CHO.

**Figure 2:**
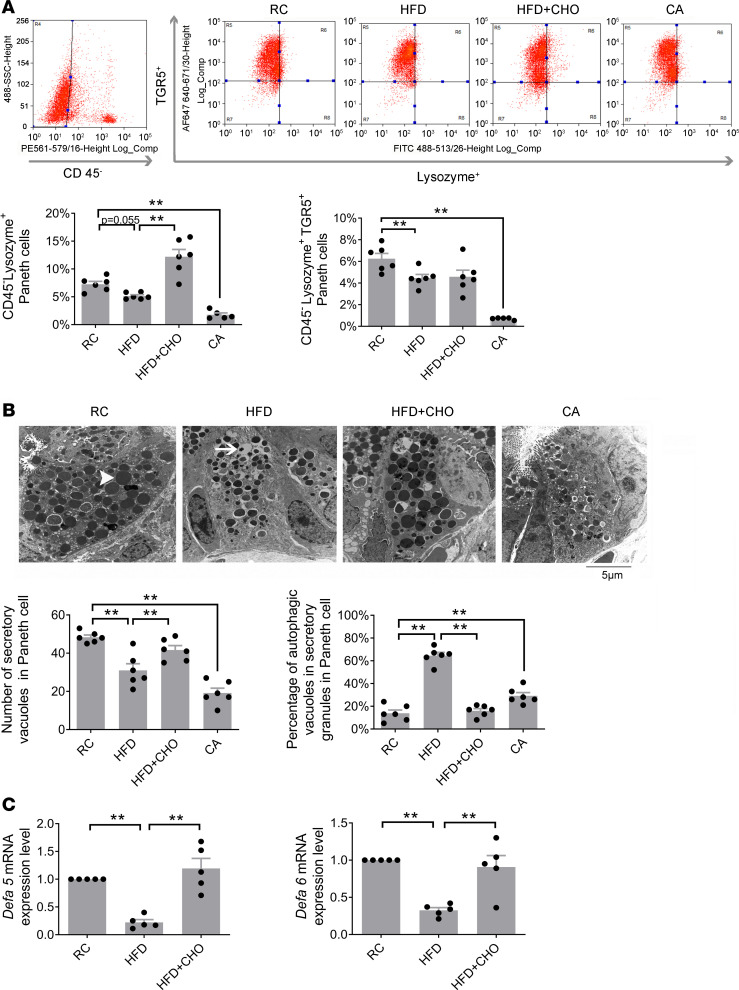
HFD induces a decrease in Paneth cells and antimicrobial peptide prevented by cofeeding with cholestyramine. (**A**) HFD induced a reduction in lysozyme^+^ Paneth cells (*n* = 6, *P* = 0.055) as well as lysozyme^+^TGR5^+^ Paneth cell expression (*n* = 6) in the crypts. Administration of cholestyramine prevented this decrease in lysozyme^+^ Paneth cells (*n* = 6). Concurrent oral cholic acid also induced a significant decrease in both lysozyme^+^ Paneth cells or lysozyme^+^TGR5^+^Paneth cell (*n* = 5–6). (**B**) HFD causes a decrease in Paneth cell secretory granules. Transmission electron micrograph of small intestinal Paneth cells showed a 34% and 53% decrease in Paneth cell secretory granules (white arrowhead) from rats fed a HFD and cholic acid diet, respectively (*n* = 6, **P < 0.01). An increase in percentage of secretory granules with vacuoles (white arrow) was also observed in Paneth cell from rats fed a HFD, which was prevented by cofeeding with cholestyramine (*n* = 6). A similar increase in vacuoles was observed in rats given cholic acid (*n* = 6). Scale bar: 5 μm. (**C**) Reduction in gene expression of α-defensin 5 (*Defa5*) and 6 (*Defa6*) in rats given a HFD. A 68% and 67% decrease in gene expression of Defa5 and Defa6, respectively, in the crypts of ileum in rats fed a HFD (*n* = 5). These decreases in *Defa5* and *Defa6* gene expression were prevented by concurrent oral feeding with cholestyramine (*n* = 5). CHO, cholestyramine. All data are shown as mean ± SEM. *P* values were determined by 1-way ANOVA. **P < 0.01.

Using CD45 as a hematopoietic cell marker, Paneth cells were identified as a CD45^−^lysozyme^+^ cell fraction. Flow cytometry showed a reduction in lysozyme^+^ Paneth cells (*n* = 6, *P* = 0.05; Figure 2A) as well as lysozyme^+^TGR5^+^ Paneth cell expression (*n* = 6, *P* < 0.01; Figure 2A) in the crypts after HFF or oral CA feeding (*n* = 5–6, *P* < 0.01; Figure 2A). Administration of CHO prevented this decrease in lysozyme^+^ Paneth cells although it did not affect the fraction expressing TGR5 (*n* = 6, *P* < 0.01; Figure 2A).

Transmission electron micrograph of Paneth cells showed a loss of electron-dense secretory granules and an increase in the percentage of granules with vacuoles in the ileum of rats given a HFD (Figure 2B) or rats fed RC supplemented with CA (Figure 2B). These changes were prevented by cofeeding with CHO in rats given a HFD (Figure 2B).

RT-qPCR studies demonstrated that HFF caused a 68% and 67% decrease in gene expression of α-defensin 5 (*Defa5*) and α-defensin 6 (*Defa6*) in the crypts of ileum, respectively (*n* = 5, both *P* < 0.01, Figure 2C). These changes were prevented by concurrent feeding with CHO (*n* = 5, both *P* < 0.01, Figure 2C). A similar reduction in *Defa5* and *Defa6* was observed in rats fed with CA (data not shown).

### HFF induces protein and gene expression of ER stress, autophagy, and DNA damage.

**Figure 3:**
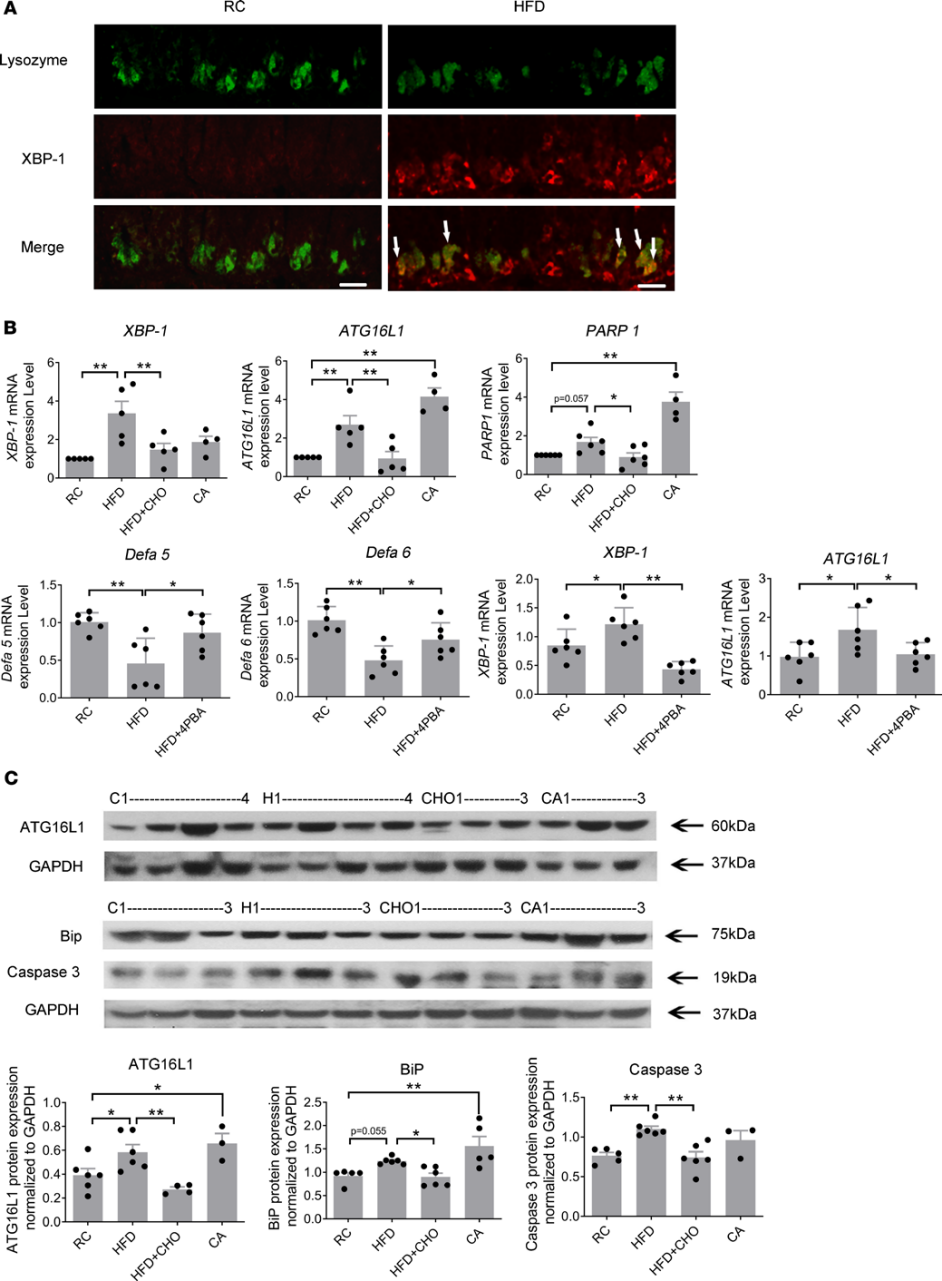
HFD induces protein and gene expression of ER stress, autophagy, and DNA damage. (**A**) Immunocytochemical staining of ileal specimen from rats fed regular chow (RC) or HFD (lysozyme, green; XBP-1 [ER stress marker], red). HFD induces increased expression of XBP-1 in lysozyme^+^ Paneth cells. Scale bar: 50 μm. (**B**) High-fat feeding caused an increase in gene expression of *XBP-1* and *ATG16L1* in the crypts of ileum (*n* = 5). These increases in *XBP-1* and *ATG16L1* gene expression were prevented by concurrent oral feeding with cholestyramine (*n* = 5). Oral feeding with cholic acid caused a similar increase in *ATG16L1* and *PARP1* gene expression (*n* = 4–5). HFD induced alteration of α-defensins, autophagy, and ER stress were prevented by an ER stress inhibitor (4BPA) (*n* = 6). (**C**) Western blot showing the density of ATG16L1 (autophagy marker), BiP (ER stress marker), and caspase-3 (DNA damage marker) immunoreactive bands at 60 kDa, 75 kDa, and 19 kDa, which were observed in the ileal crypts from rats fed RC. HFD caused a significant increase in ATG16L1 and caspase-3–immunoreactive bands (*n* = 5–6). Oral feeding with cholic acid caused a similar increase in ATG16L1 and BiP expression (*n* = 3–6). These increases in BiP, ATG16L1, and caspase-3 expression in response to HFD were prevented by concurrent oral feeding with cholestyramine (*n* = 4–6). Part of the membrane used for protein expression studies of TGR5 in Figure 1E was used for Western blotting of ATG16L1. Hence, the 2 blots share the same GAPDH. C, RC; H, HFD; CHO, HFD + cholestyramine; CA, cholic acid. *P* values were determined by 1-way ANOVA. *P < 0.05, **P < 0.01.

Using X-box–binding protein 1 (XBP-1) as an ER stress marker, we performed immunohistochemical studies and showed that XBP-1 expression was increased at the bottom of ileal crypts of rats given a HFD (Figure 3A). RT-qPCR studies showed increased gene expression of *XBP-1* and autophagy-related protein 16-1 (*ATG16L1*, an autophagy marker) in the crypts of ileum after 2 weeks of HFF (*n* = 5, both *P* < 0.01). The gene expression of *ATG16L1* and poly ADP ribose polymerase (*PARP*, an apoptotic marker) increased in rats fed CA (*n* = 4–5, *P* < 0.01; Figure 3B). Concurrent feeding with CHO prevented these increases in *XBP-1*, *ATG16L1*, and *PARP1* gene expression (*n* = 5–6, *P* < 0.05 or *P* < 0.01; Figure 3B). To demonstrate that these cellular changes in Paneth cells are due to ER stress, we showed that administration of 4-phenylbutyrate (4PBA), an ER stress inhibitor, normalized α-defensin expression and prevented induction of ER stress and autophagy caused by HFF (*n* = 6, *P* < 0.05 or *P* < 0.01; Figure 3B).

Western blot showed that the density of ATG16L1- (autophagy marker), BiP- (ER stress marker), and caspase-3–immunoreactive (cell apoptosis marker) bands at 60 kDa, 75 kDa, and 19 kDa, respectively, were observed in the ileal crypts of rats fed RC. After HFF there were significant increases in ATG16L1- and caspase-3–immunoreactive bands (*n* = 5–6, *P* < 0.05 or *P* < 0.01; Figure 3C). Oral feeding with CA caused a similar increase in BiP and ATG16L1 expression (*n* = 3–6, *P* < 0.05 or *P* < 0.01; Figure 3C). These increases in BiP, ATG16L1, and caspase-3 expression in response to HFF were prevented by concurrent feeding with CHO (*n* = 4–6, *P* < 0.05 or *P* < 0.01; Figure 3C).

### Ex vivo study (intestinal explants).

**Figure 4:**
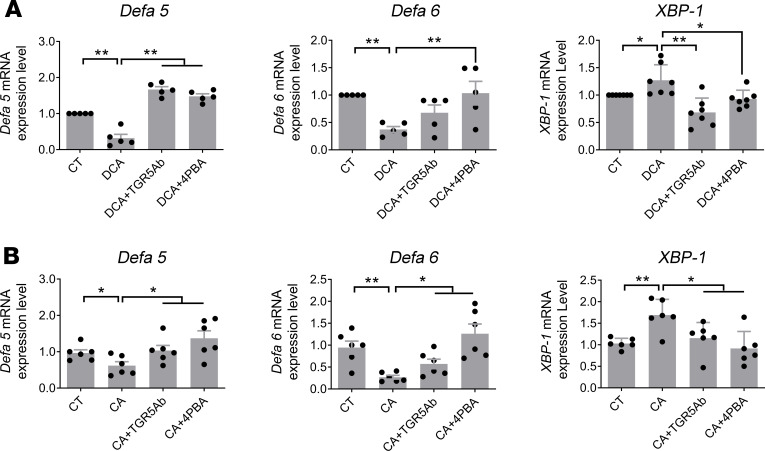
DCA and CA increase ER stress and reduction of α-defensins in cultured ileum (in vitro). (**A**) DCA (100 μM) and (**B**) CA (100 μM) caused a reduction in α-defensin and ER stress in whole-mount cultured ileum. Pretreatment with TGR5 antibody (4 μg/mL) or 4PBA (10 mM) prevented the DCA- or CA-induced alteration of α-defensins and ER stress (*n* = 5–7). *P* values were determined by 1-way ANOVA. *P < 0.05, **P < 0.01.

To provide direct evidence that bile acids induce damage in Paneth cells, we showed that there was a decrease in *Defa5* and *Defa6* gene expression and an increase in *XBP-1* gene expression in whole-mount ileal tissue incubated with DCA (100 μM) (Figure 4A) or CA (100 μM) (Figure 4B) for 24 hours. These alterations were prevented by TGR5 antibody (4 μg/mL) or by 4PBA (10 mM). However, administration of bile acid nuclear receptor, farnesoid X receptor (FXR) agonist GW4064 did not affect the expression of *Defa5*, *Defa6*, *XBP-1*, and *ATG16L1* in intestinal explants (data not shown).

### HFF alters bacterial community composition of the ileal mucosa, which is prevented by CHO or 4PBA.

**Figure 5:**
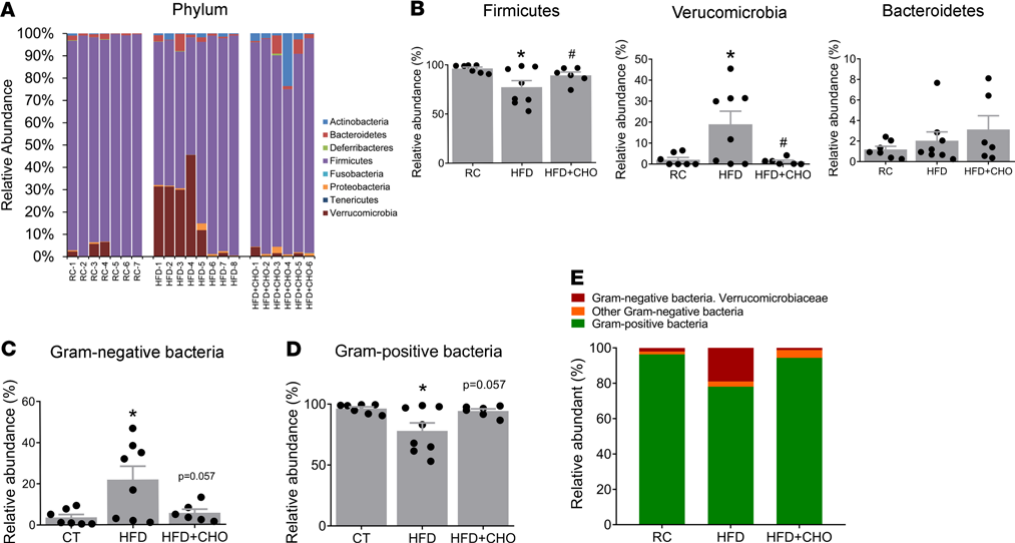
HFD-induced dysbiosis at the phylum level in the ileum mucosa is prevented by cholestyramine. (**A**) Relative abundance of OTUs classified at the level of phylum. (**B**) Relative abundance of Firmicutes, Verrucomicrobia, and Bacteroidetes. (**C** and **D**) Relative abundance of (**C**) Gram-negative and (**D**) -positive communities. (**E**) Among Gram-negative bacteria, Verrucomicrobia (*Akkermansia muciniphila*) was increased by 9-fold (percentage of total bacteria). These changes were prevented by concurrent feeding with CHO. (*n* = 6–8 per group). CHO, cholestyramine. *P* values were determined by 1-way ANOVA test. *P < 0.05 versus RC; ^#^ *P* < 0.05 versus HFD.

**Figure 6:**
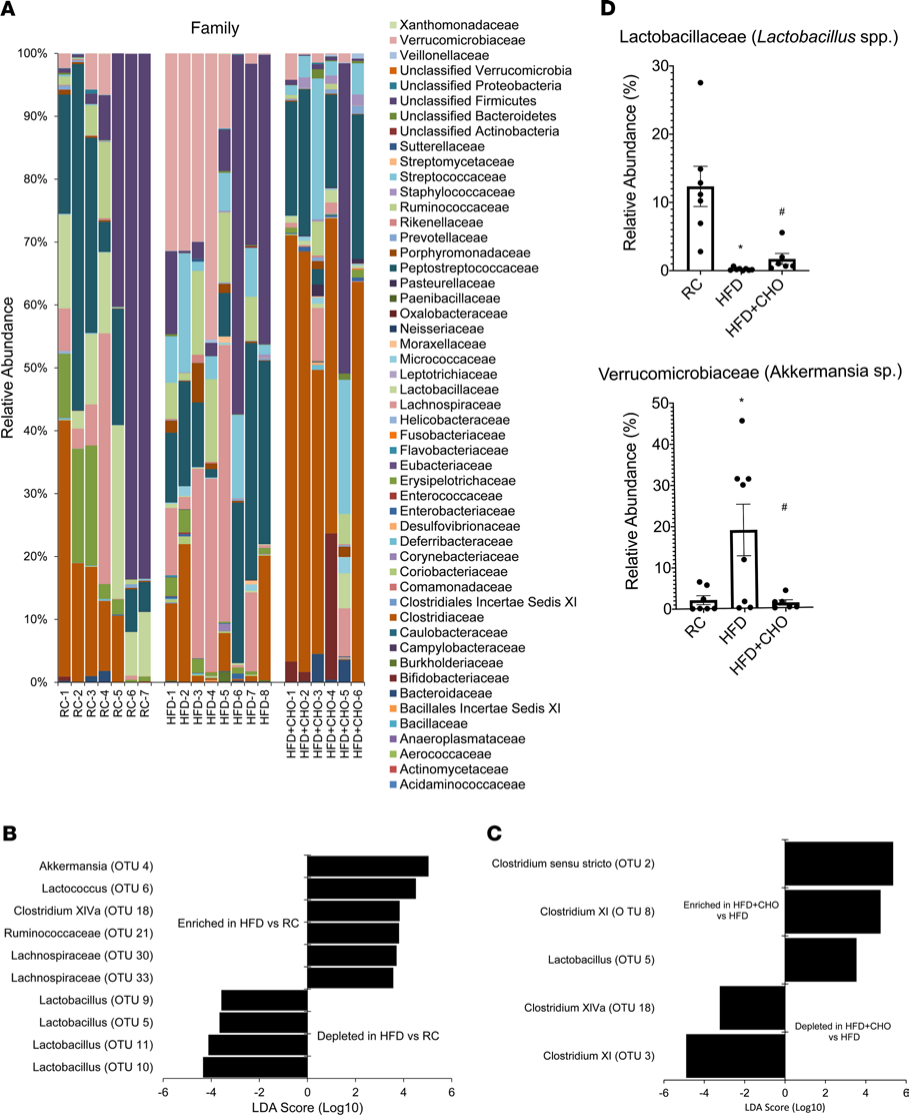
HFD-induced dysbiosis in the ileum mucosa is prevented by cholestyramine. (**A**) Relative abundance of OTUs classified at the level of family. (**B** and **C**) OTUs were significantly depleted or enriched after HFD (LEfSe, *n* = 7 or 8 per group). (**D**) As a group, the relative abundance of Lactobacillaceae and Verrucomicrobiaceae was altered after HFD+CHO compared with HFD (*n* = 6 or 8 per group). *P* values were determined by unpaired 2-tailed Student’s *t* test (2 groups) or by 1-way ANOVA (more than 2 groups). *P < 0.05 versus RC; **^#^***P* < 0.05 versus HFD. CHO, cholestyramine.

Compared with controls, there was a significant reduction in the relative abundance of Firmicutes and an increase in Verrucomicrobia after HFF (Figure 5, A and B). An increase in the relative abundance of Gram-negative bacteria after HFF and reduction of Gram-positive bacteria were observed (Figure 5, C and D). Among the Gram-negative bacteria, Verrucomicrobia (*Akkermansia muciniphila*) was significantly increased (Figure 5, B and E). Bacteroidetes showed an increase, but it did not reach statistical significance (Figure 5B). Specific taxa affected by HFF included a reduction in 4 taxa identified as *Lactobacillus* as well as enrichment in other operational taxonomic units (OTUs), such as *Clostridium XIVa*, Ruminococcaceae, Lachnospiraceae, and *Akkermansia* (linear discriminant analysis effect size [LEfSe], *P* < 0.05, Figure 6, A and B). HFF resulted in a significant depletion in the relative abundance of Lactobacillaceae from 12.4% to 0.2% and an increase in the abundance of Verrucomicrobiaceae from 2.1% to 18.9% (Figure 6D).

Concurrent oral feeding with the bile acid binder CHO significantly increased the relative abundance of Lactobacillaceae while depleting Verrucomicrobiaceae to baseline levels (Figure 6D). Further, *Clostridium* XI, *Clostridium* XIVa, and *Akkermansia* were significantly depleted, while *Lactobacillus*, *Clostridium* XI, and *Clostridium* sensu stricto were enriched in HFD+CHO when compared with HFD (LEfSe, *P* < 0.05; Figure 6, B–D).

**Figure 7:**
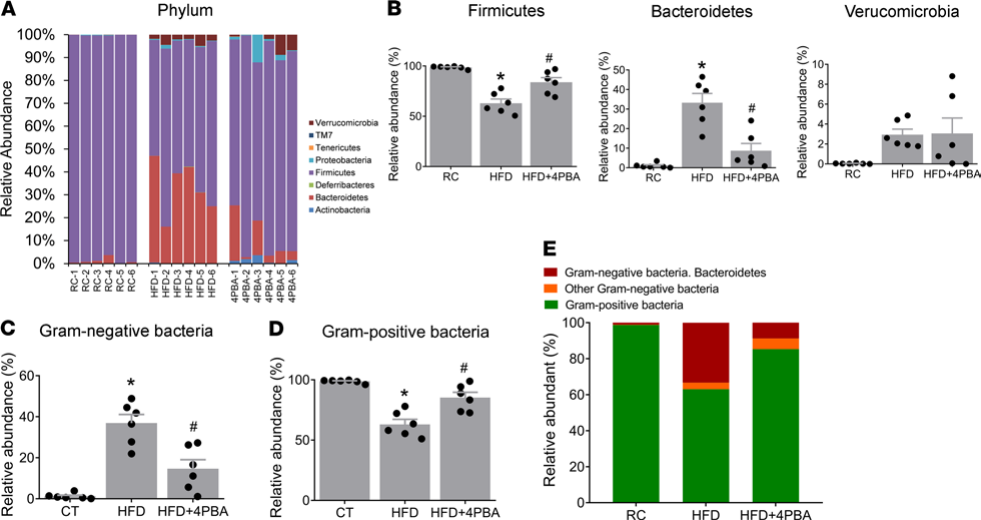
HFD-induced dysbiosis at phylum level in the ileum mucosa is prevented by 4PBA. (**A**) Relative abundance of OTUs classified at the level of phylum. (**B**) Relative abundance of Firmicutes, Bacteroidetes, and Verrucomicrobia. (**C** and **D**) Relative abundance of (**C**) Gram-negative and (**D**) -positive communities. (**E**) Among Gram-negative bacteria, Bacteroidetes was increased by 31.62-fold, from 1.05% ± 0.50% to 33.28% ± 4.75% (percentage of total bacteria) (*n* = 6 per group). These changes were prevented by 4PBA (ER stress inhibitor). *P* values were determined by 1-way ANOVA. *P < 0.05 versus RC; ^#^*P* < 0.05 versus HFD.

**Figure 8:**
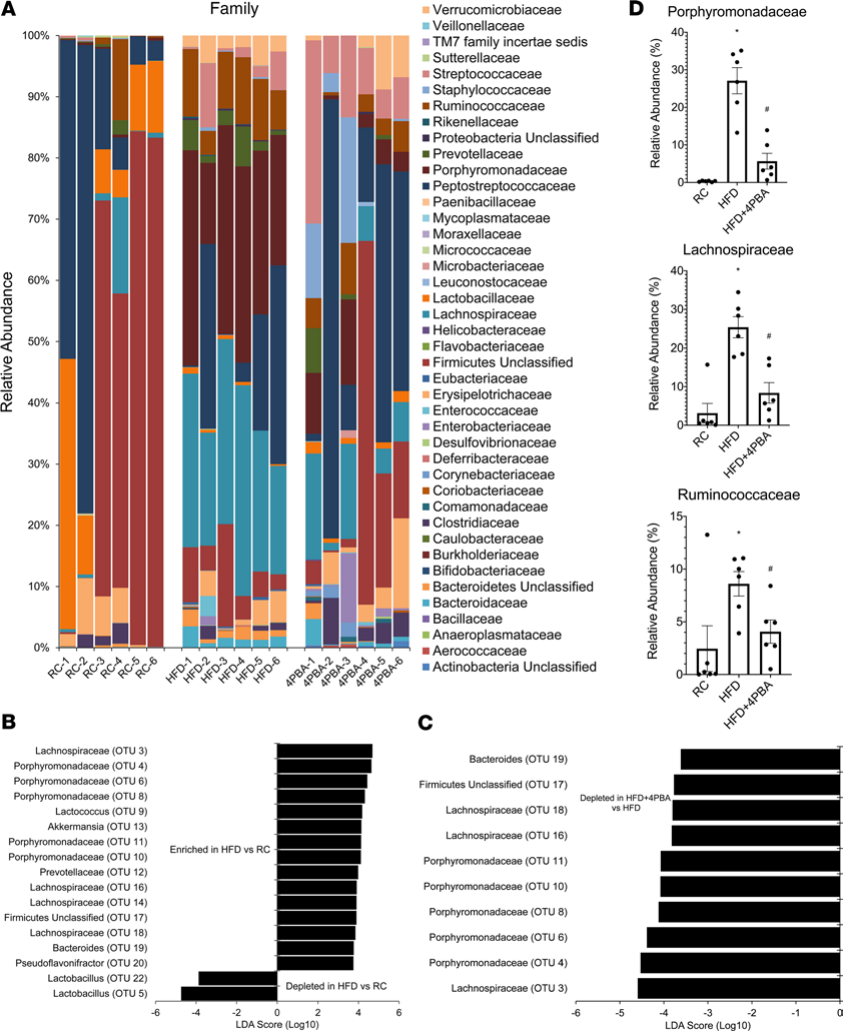
HFD-induced dysbiosis in the ileum mucosa is prevented by 4PBA. (**A**) Relative abundance of OTUs classified at the level of family. (**B** and **C**) OTUs were significantly depleted or enriched after HFD (LEfSe, *n* = 6 per group). (**D**) As a group, the relative abundance of Porphyromonadaceae, Lachnospiraceae, and Ruminococcaceae was altered after HFD+4PBA compared with HFD. *P* values were determined by unpaired 2-tailed Student’s *t* test (2 groups) or by 1-way ANOVA (more than 2 groups). *P < 0.05 versus RC; **^#^** *P* < 0.05 versus HFD.

In a separate group of rats, gut microbial changes induced by 2 weeks of HFF were prevented by an inhibitor of ER stress, 4PBA (Figures 7 and Figure 8, A–C). Two OTUs, identified as *Lactobacillus*, were significantly depleted after HFF (Figure 8B). A number of OTUs were significantly enriched after HFF, including Lachnospiraceae, *Akkermansia*, Porphyromonadaceae, *Bacteroides*, and unclassified Firmicutes (LEfSe, *P* < 0.05, Figure 8B). As a group, the relative abundances of Porphyromonadaceae, Lachnospiraceae, and Ruminococcaceae significantly increased from 0.32% to 27.1%, 3.1% to 25.4%, and 2.5% to 8.6%, respectively, after HFF (Figure 8D).

Concurrent treatment with 4PBA significantly decreased the relative abundance of the Porphyromonadaceae, Lachnospiraceae, and the Ruminococcaceae (Figure 8D). Furthermore, Bacteroides, unclassified Firmicutes, Lachnospiraceae, and Porphyromonadaceae were all significantly depleted in HFD+4PBA when compared with HFD (LEfSe, *P* < 0.05, Figure 8C).

### HFD-associated gut metagenome is altered with genes involved in amino acid and energy metabolism.

**Figure 9:**
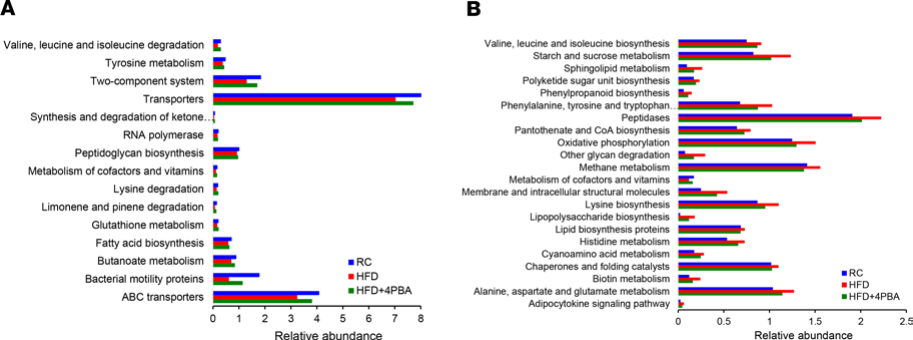
HFD altered genes from microbial community, which was prevented by 4PBA. (**A**) The relative abundance of metabolic gene pathways was altered in HFD group. The abundance of metabolic gene pathways was analyzed using PICRUSt and is based on the 16S rRNA sequencing data. Only the relative gene pathway abundances that were identified as being significantly different for RC, HFD, and HFD+4PBA are shown (*P* < 0.01, for all pairwise comparisons, by 1-way ANOVA). Note that, genes for Gram-positive cell wall synthesis (peptidoglycan biosynthesis) were decreased in the HFD group, and this was reversed by 4PBA treatment. (**B**) Compared with the regular chow (RC), HFD increased many genes, mainly, for amino acid and energy metabolism. Genes responsible for synthesis of the Gram-negative cell wall (lipopolysaccharide biosynthesis, lipopolysaccharide biosynthesis proteins) were enriched in the HFD group, and this was also reversed by 4PBA.

To better understand the biological significance of the alteration in the gut bacterial communities induced by HFF and 4PBA, we investigated the metabolic features of the resulting communities using phylogenetic investigation of communities by reconstruction of unobserved states (PICRUSt). PICRUSt is a prediction tool for inferring the potential metabolism of a given bacterial community from an OTU table using precomputed Kyoto Encyclopedia of Genes and Genomes (KEGG) pathway reference profiles. Compared with RC, HFD resulted in enrichment of many genes involved in amino acid and energy metabolism (Figure 9). There was a significant reduction (*P* < 0.01) in the expression of genes for Gram-positive cell wall synthesis (peptidoglycan biosynthesis) and an enrichment of genes responsible for synthesis of the Gram-negative cell wall (lipopolysaccharide biosynthesis) in response to HFD. These changes were prevented by 4PBA (Figure 9). These potential metabolic changes are in agreement with the gut microbiome composition alterations showing enrichment of Gram-negative bacteria following 2 weeks of HFF, and these changes were prevented by treatment with 4PBA.

## Discussion

Although there is accumulating evidence that HFF may alter the gut microbial composition, the mechanisms leading to these changes are not clearly understood. In this study, we demonstrated that TGR5 receptors are expressed by Paneth cells in intestinal ileal crypts. Rodents fed HFD or RC supplemented with CA had significantly increased fecal and serum bile acid levels, increased TGR5 protein expression, and decreased numbers of intestinal Paneth cells. This was accompanied by markers of Paneth cell dysfunction, including subcellular abnormalities in Paneth cell secretory granules, decreased expression of Paneth cell–specific α-defensins (*Defa5* and *Defa6*), and enhanced ER stress and autophagy.

We found that an important mucosal microbial change associated with HFF is an increase in Gram-negative bacteria and corresponding decrease in Gram-positive bacteria. These were accompanied by phyla-level changes, including a decreased abundance of Firmicutes and a relative enrichment in Bacteroidetes and Verrucomicrobia. Our results differ from those of prior studies that have documented an increased abundance of Firmicutes and decreased relative proportion of Bacteroidetes in response to a HFD (28, 29). However, these prior studies mainly evaluated fecal samples representative of the distal gut microbiota, while regional variations in environmental conditions, such as pH, oxygen availability, and nutrient availability, promote differences in gut microbial ecology in different regions of the gastrointestinal tract (30). Similar to our findings, Lecomte et al. reported a decrease in abundance of Gram-positive Firmicutes and an increase in Gram-negative Bacteroidetes after HFF (29). These investigators used samples collected from the cecum, which is more closely aligned with our approach. Moreover, as opposed to luminal bacterial communities, which are probably more important for nutrient digestion, we analyzed changes in the mucosal-associated microbiota, which are intimately associated with the host epithelium and are likely more relevant in studies of host-microbial immune signaling (31) and mucosa permeability. We noted that *Akkermansia muciniphila*, a genus in the phylum of Verrucomicrobia, was significantly increased following HFF (Figure 5B). It is well known that HFF is associated with impaired intestinal permeability and mucosal inflammation (32, 33). *Akkermansia muciniphila* is a Gram-negative anaerobe and a mucin degrader that uses mucin as nutrients. Thinning of the mucus layer may weaken gut barrier function and enhance transepithelial migration of microbes and their metabolites. This, in turn, predisposes the host to develop metabolic syndrome, which is common among subjects on a HFD (34).

In addition to phylum-level changes, we also observed depletion of certain OTUs, such as *Lactobacillus*, while certain OTUs were enriched, including *Clostridium* XIVa group, Ruminococcaceae, Lachnospiraceae, and *Akkermansia* *muciniphila* in rats receiving HFD. These changes are likely due to dysfunction of the Paneth cell; however, local factors may also contribute to alterations in microbial composition in the ileum. The mucosal microenvironment may favor specific microorganisms, including known butyrate producers *Clostridium* XIVa cluster, Ruminococcaceae, and Lachnospiraceae families, compared with the luminal space (31, 35). Moreover, the presence of bile acids places a major selective pressure on the microbial community structure in the gut, with Gram-negative bacteria showing higher tolerance to bile acids compared with Gram-positives bacteria (12). Prior studies have demonstrated that diets supplemented with CA-enriched bacteria possessing 7α-dehydroxylation activity, including members of *Clostridium* XIVa group, Ruminococcaceae, and Lachnospiraceae (7, 9). Another study showed a significant and positive correlation between secondary bile acid concentration in feces and abundance of members of the *Clostridium* XIVa cluster (36). Our results also support this and suggest that HFD and bile acids promote expansion of the *Clostridium* XIVa cluster. Meanwhile, Floch et al. reported that bile acids appear to be toxic to *Lactobacillus* in a dose-dependent manner (37). Reduction in the abundance of *Lactobacillus*, a major source of bile salt hydrolase activity in the gut, may result in increased tauro-β-muricholic acid levels, inhibition of intestinal FXR signaling, and further increased bile acid pool size (38). Taken collectively, this suggests that a HFD results in increased bile acids, which may cause ileal dysbiosis via direct microbial actions as well as inducing Paneth cell dysfunction.

To demonstrate that taxonomic shifts are mediated by bile acids, rats were fed either a diet of RC supplemented with CA or a HFD mixed with CHO to decrease the bile acid pool (39, 40). Murine animals fed normal chow mixed with CA demonstrated similar taxonomic shifts as those provided with a HFD (7, 9). In contrast, administration of CHO along with a HFD to rats resulted in normalization of microbial composition similar to that of rats receiving RC. Specifically, Gram-positive and Gram-negative bacteria levels normalized as did the relative abundance of Firmicutes and Verrucomicrobia and *Lactobacillus* and *Clostridium* XIVa cluster after administration of CHO. These data support the notion that bile acids promote changes in the gut microbial ecology and that these changes can be reversed by administration of a bile acid sequestrant.

It is interesting that the bile acid binder CHO produced only a modest recovery of Lactobacilli following HFF. This may be due to the fact that Lactobacilli are highly sensitive to the inhibitory actions of bile acids. Both DCA and chenodeoxycholic acid show inhibitory effects on Lactobacilli at a concentration as low as 0.67 mM (37). Hence, the small amount of bile acids remaining in the lumen that are not bound by CHO may be sufficient to inhibit the growth of Lactobacilli.

Murakami et al. reported that a HFD resulted in increased colonic bile acid concentration and provided evidence of epithelial barrier dysfunction (41). Gut dysbiosis likely causes this permeability impairment secondary to Paneth cell dysfunction. In the presence of HFD or RC supplemented with CA, there is upregulation of TGR5 expression and evidence of Paneth cell dysfunction marked by loss of secretory granules, decreased expression of α-defensins, and markers of ER stress and autophagy. Paneth cells are known to directly sense and regulate host-microbial interactions through MyD88-dependent Toll-like receptor activation (24). Activation of Paneth cells results in release of antimicrobial peptides that promote integrity of the gut epithelium. However, Paneth cells only sense bacteria closely associated with the mucosal surface; they are relatively insensitive to luminal bacterial populations. This may be due to the fact that antimicrobial factors secreted by Paneth cells are largely compartmentalized within the mucus layer covering the intestinal epithelium (42). These observations may likely explain the differential effects of bile acids on mucosal versus luminal bacteria composition in the ileum.

We next performed ex vivo studies to provide evidence that bile acids act directly on Paneth cells. Incubation of ileal explants with DCA or CA resulted in a significant decrease in gene expression of *Defa5* and *Defa*6. Similarly, prior studies have also reported decreased expression of antimicrobial peptides by Paneth cells in response to HFD. Guo et al. demonstrated that HFD in mice resulted in alteration in the gut microbial composition accompanied by reduced antimicrobial peptides lysozyme and Reg IIIγ (43). Similarly, Lee and colleagues reported that mice fed a HFD had decreased Paneth cells, reduced lysozyme release, and disruption of intestinal barrier function and were more susceptible to experimentally induced colitis (44). These data suggest that a HFD is detrimental to Paneth cell function, which results in reduced antimicrobial peptide release. Our study demonstrates that harmful action of HFD is mediated by bile acids acting directly on Paneth cells.

In order to determine the mechanisms by which bile acids induce Paneth cell injury and dysfunction, we demonstrated that HFF significantly increased gene expression of markers of ER stress response (*XBP-1*) as well as a marker of autophagy (*ATG16L1*). We confirmed that upregulation of gene expression was occurring in Paneth cells by immunohistochemistry. Protein expression of ER stress marker (BiP) was enhanced in vivo when rats were fed RC supplemented with CA, and gene expression of *XBP-1* was upregulated in ileal explants when they were cultured in the presence of DCA or CA. On the other hand, gene expression normalized when rats were fed a HFD along with CHO. Similarly, gene expression of XBP-1 was normalized in vitro in the presence of TGR5 antibodies or 4PBA. Moreover, we also showed that inhibition of ER stress response by 4PBA could reverse community-level changes in gut microbial composition. This suggests that bile acid–induced Paneth cell dysfunction plays a major role in gut dysbiosis in the ileal mucosa induced by HFD. By virtue of their highly secretory function, Paneth cells are characterized by extensive networks of ER and thus are particularly susceptible to increased ER stress response and autophagy (45). Prior reports have demonstrated that a HFD and obesity induce ER stress in different organ systems, including adipose tissue, the amygdala, and the thyroid gland (46–48). Guo et al. reported increased mRNA expression of ER stress markers, including *BiP*, activating transcription factor 4 (*ATF4*), and c/EBP-homologous protein (*CHOP*) in mice fed a HFD (49). Meanwhile, Hodin et al. demonstrated an inverse correlation between Paneth cell antimicrobial peptides and measures of ER stress, such as BiP and ATF4 levels, in obese individuals (27). Taken collectively, these data indicate that HFD has detrimental effects on Paneth cells. Our studies demonstrated that these abnormalities are mediated by the TGR5 receptor, which activates ER stress responses and autophagy. These events result in Paneth cell dysfunction, reduced expression of antimicrobial peptides, and subsequent gut dysbiosis in the ileal mucosa. Furthermore, this compositional shift in the gut microbial microenvironment is characterized by overabundance of certain taxa that are geared toward increasing the bile acid pool size, which may result in further deleterious changes in Paneth cell function. Although bile acids may also act via the bile acid nuclear receptor (FXR), we demonstrated that FXR agonists do not affect gene expression of antimicrobial peptides by Paneth cells. This suggests that TGR5 is the main pathway by which HFF induces alterations in Paneth cell function.

A defect in Paneth cell numbers and antimicrobial peptide production has been found in certain gastrointestinal disorders, such as Crohn’s disease (50, 51) and necrotizing enterocolitis (52), leading to intestinal dysbiosis. Recently, Günther and colleagues (53) reported that patients with Crohn’s disease had significant increases in interferon λ, accompanied by severe ileal inflammation and loss of Paneth cells. In Crohn’s disease, the decline of Paneth cell appears to be associated with caspase-8–mediated programmed necrosis (53). On the other hand, in our studies we observed that caspase-3 was increased following HFF or oral feeding with CA. This suggests that apoptosis is a major pathway leading to the loss of Paneth cells in bile acid toxicity. We did not examine caspase-8 and cannot rule out its participation in the decrease of Paneth cell following HFF.

Our studies have some limitations. To provide conclusive evidence that bile acid toxicity on Paneth cells contributes to gut dysbiosis we need a Paneth cell–specific TGR5-knockout rodent model. In the absence of such a genetic model, we demonstrated that the bile acid binder CHO reversed the effects on gut dysbiosis induced by HFF. This suggests that ileal mucosal dysbiosis is likely mediated directly or indirectly by bile acids. We also showed that an inhibitor of ER stress given systemically prevented dysbiosis following HFF, suggesting that alteration in ileal microbial composition may result from metabolic malfunctioning of the host immune system, such as Paneth cells. While this interpretation is logical, it may be subject to criticism. CHO is an anion exchange resin that binds more than just bile acids. The ER stress inhibitor 4PBA may act on a number of cell types other that Paneth cells. Hence, further studies using tissue-specific TGR5-knockout models are needed to confirm our hypothesis.

**Figure 10:**
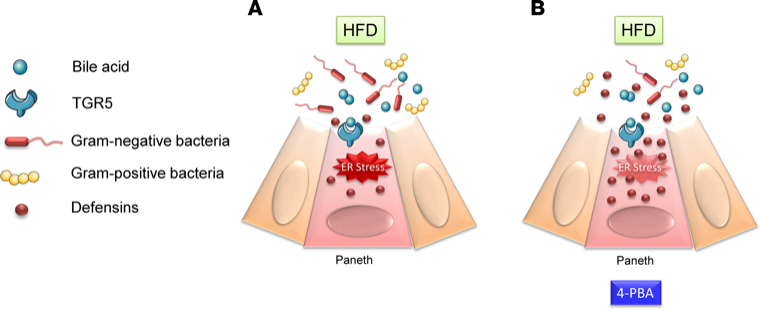
A schematic diagram depicting the mechanism by which bile acids cause gut dysbiosis through Paneth cells. (**A**) HFD lead to increased bile acid production and high concentrations of DCA. The activation of TGR5 by DCA causes ER stress, autophagy, and reduction of α-defensins in Paneth cell, which favors the growth of Gram-negative bacteria and results in mucosal microbial dysbiosis. (**B**) These changes are prevented by 4PBA.

In conclusion, we propose that HFF leads to increased bile acid production and upregulation of TGR5 receptors in Paneth cells. Overactivation of TGR5 by bile acids causes ER stress, resulting in autophagia and reduction of secretion of defensins into the intestinal crypts. This creates an environment favoring the growth of Gram-negative bacteria adjacent to the ileal epithelium. Paneth cell injury can be prevented by inhibition of ER stress, such as the use of 4PBA, to restore normal production of defensins and prevents dysbiosis occurring adjacent to the ileal mucosa (Figure 10). Further studies may focus on TGR5 as a potential therapeutic target for obesity and metabolic-related conditions.

## Methods

### Materials

The chemicals used in this study are described in detail in the Supplemental Methods (supplemental material available online with this article; https://doi.org/10.1172/jci.insight.138881DS1).

### Animal preparation

Experiments were performed on male Sprague-Dawley rats (100–150 g) (Charles River Laboratories). Rats were housed 3 per cage in a pathogen-free facility maintained on a 12-hour light/dark cycle. They were given food and water ad libitum and allowed to acclimate to the facility for 2 days before being randomly assigned to dietary treatments.

Rats were fed for 2 consecutive weeks with the following: (a) a control diet of RC (11.3% kcal from fat), (b) a HFD (58% kcal from fat, D12330, Research Diets), (c) a HFD mixed with 6% (w/w) CHO (bile acid binder C4650, MilliporeSigma), or (d) RC mixed with CA (0.051% C1129, MilliporeSigma).

In a separate group, rats were fed for 2 consecutive weeks with the following: (a) a control diet of RC, (b) a HFD, (c) HFD+4PBA (MilliporeSigma) (100 mg/kg/d, i.p.).

At the end of the 2-week period, rats in each group were food deprived for 12 hours and then euthanized. Fresh serum and fecal samples were collected after a 12-hour fast and stored at –80°C for analysis of bile acid concentration. Ileal tissue and crypts were harvested for immunochemistry, flow cytometry, MiSeq analysis of bacteria, Western blot, RT-qPCR, electron microscopy and ileal explant culture studies.

### Total bile acid concentration measurement

Total bile acid from fecal samples and serum were measured enzymatically using the Rat Total Bile Acids Assay Kit (Crystal Chem). Fecal samples were homogenized in 75% ethanol and incubated for 2 hours at 50°C. The extract was centrifuged at 13,000*g* for 10 minutes, and fecal supernatant was loaded for determination of absorbance at 540 nm, according to the manufacturer’s instruction (54, 55).

### Illumina MiSeq analysis of bacteria

#### MiSeq Illumina sequencing.

Samples were processed using the MiSeq Illumina sequencing platform. 16S rRNA gene libraries were constructed using primers specific to the V4 region (see Supplemental Methods).

#### OTU assignment.

Sequences were curated using mothur (v.1.40) (56) following the steps outlined in the MiSeq SOP (57). Sequences were assigned to OTUs using a 97% similarity cutoff and classified against the Ribosomal Database Project 16S rRNA gene training set (version 16) using a naive Bayesian approach with an 80% confidence threshold. Curated OTU sequence data were converted to relative abundance ± standard error of the mean. To test for OTUs that were differentially abundant, biologically consistent, and having the greatest effect size, we used LEfSe (58). Only OTUs with a relative abundance >0.5% were considered in the LEfSe analyses.

### Prediction of the bacterial metagenome from the ileum mucosa

To predict functional potential, and better understand the biological significance of alterations in mucosal microbial communities induced by HFD and 4PBA, we analyzed the relationship between microbial structure and function associated with HFF with or without 4PBA using PICRUSt (59).

### Epithelial cell isolation, Western blot, and RT-PCR

Crypts were isolated from terminal ileum as previously described by Sato et al. (60) with some modifications. Crypts were isolated and used for RT-qPCR or Western blot studies to examine gene and protein expression of antimicrobial peptides, *Defa*5 and *Defa*6; markers of ER stress, including XBP-1, and autophagy, including ATG16L1; and poly ADP ribose polymerase (PARP). For further details, see the Supplemental Methods.

### Immunohistochemistry

Sections of ileal tissue and isolated epithelial cells were used for immunohistochemistry studies as described previously (see the Supplemental Methods) (61).

### In situ hybridization

Sections of ileal tissue were used for in situ hybridization studies as described previously (see the Supplemental Methods) (62).

### Flow cytometry

Epithelial cells were isolated as described above and then underwent flow cytometry studies as previously described (see the Supplemental Methods) (63).

### Electron microscopy

Detailed procedures for electron microscopy have been previously described (28) (see the Supplemental Methods).

Ex vivo study of ileal explant culture

To provide direct evidence that bile acids can act directly to affect Paneth cells, ileal explants were incubated with DCA (100 μM) or CA (100 μM) for 24 hours with or without TGR5 antibody or ER stress inhibitor (4PBA) to determine gene expression of α-defensins and *XBP-1* (ER stress markers) (see the Supplemental Methods).

### Statistics

All results are shown as mean ± SEM, with the number of rats indicated. For statistical analyses, differences between groups were compared by 2-tailed Student *t* test for comparisons between 2 groups or 1-way ANOVA for comparisons of more than 2 groups. *P* < 0.05 was considered significant.

### Study approval

All procedures were performed in accordance with the NIH guidelines and were approved by the University of Michigan Committee on Use and Care of Animals.

## Author contributions

CO conceived and designed research. HZ, SYZ, MG, JYL, JG, GZ, and XX obtained data from the study. HZ, SYZ, MG, and JYL analyzed and interpreted the data. HZ, SYZ, AL, and CO wrote the manuscript. CO and HZ obtained funding.

## Supplementary Material

supplemental data

## Version 1 10/15/2020

Electronic publication
