# Supplementary material for: Bile acid toxicity in Paneth cells contributes to gut dysbiosis induced by high-fat feeding
Source: JCI Insight. 2020 Oct 15;5(20):e138881. doi: 10.1172/jci.insight.138881 (PMC7605541; doi:10.1172/jci.insight.138881)
Supplement: supplemental data [file jciinsight-5-138881-s118.pdf]

Supplemental data

DNA Isolation for Illumina MiSeq analysis

Genomic DNA was extracted by using a modified protocol of the Qiagen DNeasy Blood & Tissue kit. These modifications included (i) adding a bead-beating step using UltraClean fecal DNA bead tubes (Mo Bio Laboratories, Inc.) that were shaken using a Mini-Beadbeater-16 (BioSpec Products, Inc.) for 1.5 min, (ii) increasing the amount of buffer ATL used in the initial steps of the protocol (from 180 μl to 360 μl), (iii) increasing the volume of proteinase K used (from 20 μl to 40 μl), and (iv) decreasing the amount of buffer AE used to elute the DNA at the end of the protocol (from 200 μl to 85 μl).

Crypt and epithelial cell isolation

After the rats were dissected, 4-5 cm of the terminal ileum was collected, longitudinally opened and the luminal contents were thoroughly washed with ice-cold phosphate-buffered saline (PBS). The tissue was chopped into around 5 mm pieces, and further washed with cold PBS. The tissue fragments were incubated in 30 mM ethylenediaminetetraacetic acid (EDTA)/1.5 mM dithiothreitol with PBS on ice for 20 min. After removal of EDTA medium, the tissue fragments were vigorously shaken 20 times by using a 10-ml pipette with cold PBS. The supernatant was the villous fraction and was discarded; the sediment was resuspended with cold PBS. After further vigorous vibration 50 times and centrifugation at 400 × g at 4 °C for 4 min, the supernatant was subsequently collected for crypts. Isolated crypts were centrifuged at

150–200g for 3 min to separate crypts from single cells. The final pure crypts were used for Western Blot or RT-qPCR analysis.

Dissociated crypts were centrifuged at 2,500 rpm for 5 min. The cells were washed with PBS and re-suspended in Hank's Balanced Salt Solution (HBSS, Thermo Fisher Scientific) /0.3 U/ml dispase at 37°C. Shaking was performed every 2 min for 10 min. 100 mg DNaseI was then added, and the cells were passed through a 70-mm filter (BD Bioscience). Cells were centrifuged at 2,500 rpm for 5 min and re-suspended in 4 ml HBSS with 5% FBS, followed by passage through a 100-mm filter and combination with an additional 100 mg DNaseI. Isolated cells were used for immunohistochemistry or flow cytometry studies.

Immunohistochemistry

Isolated epithelial cells were put on the slides and fixed in 4% paraformaldehyde for 15 min. Segments of the terminal ileum were fixed in 10% formalin and embedded in paraffin, sectioned at 5-μm thickness. Sections of ileal tissue or isolated epithelial cells were stained according to standard immunohistochemistry protocols and manufacturer's recommendations as described previously（52）. Double immunohistochemical staining was performed against: Lysozyme (1:250, A0099, DAKO Cytomation) and TGR5 (1:200, sc-48685, Santa Cruz Biotechnology) or Lysozyme (1:250, A0099, DAKO Cytomation) and marker of ER stress, XBP-1 (1:300, sc-32136, Santa Cruz Biotechnology), respectively. Species-specific

fluorophore-conjugated secondary antibodies Alexa Fluor 488 and Alexa Fluor 594 (Jackson ImmunoResearch Laboratories, West Grove, PA) were employed. Slides were washed with PBS and mounted with Prolong Gold Antifade reagent with DAPI (Invitrogen). Tissues were examined and photographed by use of a fluorescence microscope (Olympus BX-51, Tokyo, Japan). TGR5 and lysozyme were detected by a Leica TCS SP8 confocal laser scanning microscope (Leica TCS NT, Wetzlar, Germany).

In situ hybridization

The rats were euthanized with CO2 and perfused via the ascending aorta with phosphate buffered saline (PBS, in mmol/L: NaCl 137; KCl 2.7; Na2HPO4 10 and KH2PO4 1.8), followed by 4% paraformaldehyde in PBS. The ileum was post fixed in 4% paraformaldehyde overnight and then transferred to 20% sucrose (in PBS with 0.02 % sodium azide) for 5 days at 4 ºC. The ileum was embedded with 20% sucrose and Tissue-Tek O.C.T. (2:1) and sectioned (16 μm) using a cryostat. The sections were dried overnight at room temperature and were stored at -80 o C until further processing. Rat lysozyme and TGR5 cDNA fragments were generated by PCR from an adult rat ileum cDNA library with Eco RI and Kpn I sites incorporated up- and downstream respectively. The PCR primers for lysosyme are: forward 5'-<u>GGAATTCC</u>AGTGAACGCCTGTGGGATAC-3' (Eco RI site is underlined) , reverse: 5'-<u>GGGGTACCCC</u>GGCACAGGGCTGATGAA-3' (Kpn I site is underlined); for TGR5: forward: 5'-<u>GGAATTCC</u>ATGGCCCCAAGACCTACAAG-3',

reverse: GGGGTACCCCTGGCAAGCAGGGAGAGG-3'. The PCR products were sub-cloned into pBluescript SK, which has T3 and T7 RNA polymerase promoters, at EcoR I and Kpn I sites. (pBSK, Stratagene, La Jolla, CA). The cDNAs were confirmed by DNA sequencing. The Dig- UTP or [^{35}S]-UTP and -CTP labeled antisense and sense RNA probes were generated using standard in vitro transcription methodology. In situ hybridization was conducted using a previously described technique (53). The sections were washed 3 times with 2× SSC and then digested with 0.45 μg/ml proteinase K (Invitrogen, Carlsbad, CA) in 100 mM Tris, pH 8.0, 50 mM EDTA) for 15 minutes at 37 °C. After brief washing with distilled water, the sections were acetylated with 0.25% acetic anhydride in 0.1 M triethanolamine, pH 8.0, for 10 min. The sections were subsequently dehydrated through a graded series of ethanol.

Digoxigenin (dig)- or [^{35}S]-labeled antisense or sense probes were diluted (1:50 for dig-labeled probes, ≈ 30,000 cpm/μl for [^{35}S]-labeled probes) in hybridization buffer (50% formamide, 3 x SSC, 1 x Denhart's, 200 μg/ml yeast tRNA, 50 mM phosphate buffer, pH 7.4, 10% dextran sulfate and 10 mM DTT). 60 μl diluted probes were applied to each slide and the sections were coverslipped. Slides were then placed in sealed plastic boxes lined with filter paper moistened with 50% formamide. The boxes were wrapped with plastic wrap and incubated at 55 °C overnight.

Flow Cytometry

After epithelial cells were isolated, cells were fixed in 4% paraformaldehyde for 15

min. All staining steps were performed for 30 min at 4°C in the dark. Cells were resuspended in 100 μl sorting buffer and stained with fluorophore-conjugated antibodies as follows: with anti-CD45-PE (clone), TGR5 (1:200, sc-48685, Santa Cruz Biotechnology), Alexa Fluor 647-conjugated anti-goat IgG (Jackson). The stained cells were incubated for 1h at 4°C, subsequently washed with PBS, and re-suspended in saponin permeabilization buffer (Invitrogen, Carlsbad, CA) with Lyz-fluorescein isothiocyanate antibody (1:10, Dako, Carpinteria, CA) at room temperature (RT) for 30 min. All flow analyses were performed using a CyAn Flow Cytometer (Dako/Cytomation). All reagents and instrumentation used for flow cytometry were from BD Biosciences (San Jose, CA, USA). Hematopoietic cell exclusion was accomplished using side-scatter/forward-scatter gating that had previously been optimized to exclude all $CD45^+$ cells from identical epithelial preparations.

Western blot analysis

The Western blot study was performed as follows: Briefly, proteins extracted from ileal crypts of rats were analyzed on Ready Gel Tris·HCl (Bio-Rad Laboratories). The tissue was homogenized in ice-cold lysis buffer. The homogenate was centrifuged at 14,000 g for 10 min. Protein samples were run on Ready Gel 7.5% or 4–20% Tris·HCl for 1 h at 0.06 A and transferred to nitrocellulose Hybond-enhanced chemiluminescence membranes (GE Healthcare, Life Sciences, Pittsburgh, PA) for 1.5 h at 80 V. The membranes were blocked with StartBlock T20 blocking buffer

(Thermo Scientific, Rockford, IL) for 1 h at room temperature, probed overnight with primary antibodies against TGR5 (1:400, ab72608, Abcam, Cambridge, MA), BiP (1:1000, ab21685, Abcam, Cambridge, MA), ATG16L1 (1:1500, ab187671, Abcam, Cambridge, MA) or Caspase 3 (1:1000, ab13847, Abcam, Cambridge, MA) at 4°C overnight, and then washed in Tris-buffered saline for 1 h. The membranes were probed with corresponding horseradish peroxidase-conjugated secondary antibodies at 1:8,000 dilution for 1 h at room temperature, and the bands were visualized by electrochemiluminescence (PerkinElmer, Waltham, MA). Signals were quantified with use of Image J (National Institutes of Health, Bethesda, MD) and normalized by GAPDH.

RT-qPCR

Total RNAs were extracted from rat ileal crypts tissue by use of TRIzol reagent (Life Technologies). RNAs treated with DNase I were used to synthesize cDNA (iScript cDNA Synthesis Kit, Bio-Rad Laboratories). The resultant cDNAs were used for qPCR. The primer sets targeting *Defa*-5(PPR52884A-200), *Defa*-6 (PPR65767A), *XBP-1*(PPR54461A), *ATG16L1*(PPR50683A) and *PARP1*(PPR45370A) were purchased from QIAGEN (Valencia, CA). *GAPDH* (PPR06557B) served as an endogenous housekeeping reference gene. RT-qPCRs were analyzed by using iQ SYBR Green Supermix according to the manufacturer's instructions.

The PCR conditions were as follows: one cycle at 95°C for 10 minutes, followed by

40 two-temperature cycles at 95°C for 15 seconds and 60°C for 60 seconds. PCR amplifications were performed in a total volume of 25 µl, containing iQSYBR Green supermix (Bio- Rad Laboratories). Cytokine transcript levels were normalized to that of GADPH, and relative gene expression was expressed as the fold change (2−ΔΔCt) relative to expression in the control samples.

Electron microscopy

After rats had been sacrificed, their small intestines were immediately fixed in 1.25% glutaraldehyde, 4% formaldehyde in 0.1Mcacodylate buffer at pH7.4 at room temperature for electronmicroscopy. Next, samples were rinsed in cacodylate buffer and postfixed in 1% OsO4 in 0.067 M cacodylate buffer (pH 7.4) supplemented with 1.5% potassium ferrocyanide at 4 °C for 1 hour. After a short rinse in cacodylate buffer, dehydration was carried out in graded ethanol series, followed by embedding in Epon. After staining with uranyl acetate and lead citrate, the ultrathin sections were examined with a JEOL 1400-plus transmission electron microscope.

Ex vivo study Ileal explant culture

Ileal explant from RC rats was washed in ice-cold oxygenated phosphate-buffered saline (PBS). After incubating in ice-cold PBS containing 1% fungizone antimycotic (15290-018, GIBCO, Life Sciences Solutions) for 5 minutes, tissues were placed in a 6-well cluster with 2 mL Dulbecco's modified Eagle's medium containing 100 unit/mL penicillin–streptomycin (15070-063, GIBCO, Life Sciences Solutions).

Explants were exposed to DCA or CA (Sigma-Aldrich) at 100 μM or vehicle (0.1% ethanol) and were incubated in 37°C incubator under 95% oxygen and 5% CO2. In separate studies, explants were exposed to in the presence or absence of TGR5 antibody (4μg/ml medium; sc-48685, Santa Cruz Biotechnology) or ER stress inhibitor (4PBA, 10mmol/L, SML0309, Sigma-Aldrich, St Louis, MO). Tissues were collected after 24 hours for RT-qPCR study.
